# The ethical advantages of video conferencing in medical education

**DOI:** 10.1080/10872981.2020.1787310

**Published:** 2020-07-02

**Authors:** Joshua M. Kruger, Itay Chowers

**Affiliations:** Department of Ophthalmology, Hadassah Medical Center, Jerusalem, Israel; The Hebrew University-Hadassah School of Medicine, Jerusalem, Israel

**Keywords:** Video conferencing, -Medical Education, ethics, pharmaceutical industry, environment

## Letter to the editor

The COVID-19 pandemic has mandated drastic changes not only in the manner in which patients are treated, but also in the way that medical education and knowledge is disseminated. The risks and potential consequences of viral transmission among medical staff have necessitated replacing conventional face-to-face interactions with video conferencing [1]. Although this change may be regarded by some as troublesome or inconvenient, we believe that it can have several clear advantages.

Interactions between physicians and the pharmaceutical/biotech industry are both inevitable and important. The development of new diagnostic tools and therapeutics – and their introduction to the clinic – requires collaboration between industry and the medical establishment; however, this relationship is not limited to collaborative efforts in the development and introduction of medical innovations. Industry often make significant efforts to participate in a variety of medical curricula spanning from medical school, residency and fellowship training, and continuing medical education (CME) programs [2,3]. These opportunities can include sponsored seminars, meetings with sales representatives from pharmaceutical companies, and either direct or indirect support of CME programs.

Achieving the ideal balance with respect to fruitful collaborations between physicians and industry is not a simple task, particularly with respect to avoiding potentially harmful promotional and/or marketing activities. The pharmaceutical industry often trains and funds physicians to serve as speakers in educational programs and to give presentations either prepared or sponsored by the funding companies [4]. The pharmaceutical industry also strives to develop ‘Key Opinion Leaders’ (KOLs) who can have a significant impact on the medical community and may convey messages that are aligned with the pharmaceutical company’s agenda [5]. Indeed, studies suggest that support from industry partners can bias medical education and can influence the prescribing practices of physicians, which may not necessarily be aligned with the interests of our patients or society [6].

Attending a face-to-face conference has several advantages, including the ability to network and the opportunity to be immersed in the ‘scientific experience’ [7]. The traditional conference model also provides the opportunity to take a break from routine clinic work, thereby helping prevent burnout, which can reduce quality of care. On the other hand, traveling to a conference can be costly and time-consuming, and can have adverse environmental consequences such as increased pollution due to airline travel and other modes of transportation [8]. In addition, organizing a large scientific conference often requires a considerable financial commitment that can exceed the means of an academic institution or society [8]. The pharmaceutical industry is a common source of financial support for CME programs and conferences [2]. Although regulations designed to curb or prevent biased content in pharma-supported events have been developed, significant concerns remain with respect to the effect of industry involvement on the content and format of these meetings, as well as the eventual effects on medical education and healthcare [9].

The introduction of strict social distancing measures due to the COVID-19 pandemic has led to a global shift from face-to-face meetings, lectures, and conferences, in favor of an online meeting format. Applications and software platforms that facilitate such online meetings are now used widely among medical professionals, and resident training sessions, staff meetings, and conferences are currently conducted using these platforms. Importantly, using an online platform significantly reduces costs to within the reach of most clinical departments and scientific/clinical societies around the world. Moreover, they allow anyone with internet access – regardless of his/her nationality, location, or financial means – to attend such meetings. Lastly, online meetings eliminate the harmful environmental effects associated with large on-site conferences, with excellent economic utility.

These advantages indicate that this is an excellent opportunity to introduce the widespread usage of online meetings as a robust platform for providing unbiased medical education. Because the format requires no outside financial support, medical education can return to its roots, facilitating the open and transparent discussion of emerging therapies and technologies, ultimately benefiting clinicians, the pharmaceutical industry, and patients. After decades of having to rely – at least in part – on industry partners, the COVID-19 pandemic has led to the realization that we now have the technology on hand to facilitate a paradigm shift and return medical education to the highest ethical standards.
